# Study of the Effects of 3 h of Continuous Cardiopulmonary Resuscitation at 27°C on Global Oxygen Transport and Organ Blood Flow

**DOI:** 10.3389/fphys.2020.00213

**Published:** 2020-04-16

**Authors:** Jan Harald Nilsen, Sergei Valkov, Rizwan Mohyuddin, Torstein Schanche, Timofei V. Kondratiev, Torvind Naesheim, Gary C. Sieck, Torkjel Tveita

**Affiliations:** ^1^Anesthesia and Critical Care Research Group, Department of Clinical Medicine, UiT The Arctic University of Norway, Tromsø, Norway; ^2^Department of Research and Education, Norwegian Air Ambulance Foundation, Drøbak, Norway; ^3^Division of Surgical Medicine and Intensive Care, University Hospital of North Norway, Tromsø, Norway; ^4^Department of Physiology and Biomedical Engineering, Mayo Clinic, Rochester, MN, United States

**Keywords:** accidental hypothermia, cardiac arrest, hemodynamics, organ blood flow, cardiopulmonary resuscitation (CPR)

## Abstract

**Aims:**

Complete restitution of neurologic function after 6 h of pre-hospital resuscitation and in-hospital rewarming has been reported in accidental hypothermia patients with cardiac arrest (CA). However, the level of restitution of circulatory function during long-lasting hypothermic cardiopulmonary resuscitation (CPR) remains largely unknown. We compared the effects of CPR in replacing spontaneous circulation during 3 h at 27°C vs. 45 min at normothermia by determining hemodynamics, global oxygen transport (DO_2_), oxygen uptake (VO_2_), and organ blood flow.

**Methods:**

Anesthetized pigs (*n* = 7) were immersion cooled to CA at 27°C. Predetermined variables were compared: (1) Before cooling, during cooling to 27°C with spontaneous circulation, after CA and subsequent continuous CPR (*n* = 7), vs. (2) before CA and during 45 min CPR in normothermic pigs (*n* = 4).

**Results:**

When compared to corresponding values during spontaneous circulation at 38°C: (1) After 15 min of CPR at 27°C, cardiac output (CO) was reduced by 74%, mean arterial pressure (MAP) by 63%, DO_2_ by 47%, but organ blood flow was unaltered. Continuous CPR for 3 h maintained these variables largely unaltered except for significant reduction in blood flow to the heart and brain after 3 h, to the kidneys after 1 h, to the liver after 2 h, and to the stomach and small intestine after 3 h. (2) After normothermic CPR for 15 min, CO was reduced by 71%, MAP by 54%, and DO_2_ by 63%. After 45 min, hemodynamic function had deteriorated significantly, organ blood flow was undetectable, serum lactate increased by a factor of 12, and mixed venous O_2_ content was reduced to 18%.

**Conclusion:**

The level to which CPR can replace CO and MAP during spontaneous circulation at normothermia was not affected by reduction in core temperature in our setting. Compared to spontaneous circulation at normothermia, 3 h of continuous resuscitation at 27°C provided limited but sufficient O_2_ delivery to maintain aerobic metabolism. This fundamental new knowledge is important in that it encourages early and continuous CPR in accidental hypothermia victims during evacuation and transport.

## Introduction

Over the past years, patient case reports of accidental hypothermia with cardiac arrest (CA) indicate favorable neurologic outcome after in-hospital rewarming (Walpoth et al., 1990, 1997; Gilbert et al., 2000; Mark et al., 2012; Wanscher et al., 2012; Hilmo et al., 2014). These favorable outcomes appear to be linked to the good quality of pre-hospital emergency medical treatment provided including early start and continued cardiopulmonary resuscitation (CPR) (Monsieurs et al., 2015; Truhlar et al., 2015) in accordance to the latest international guidelines (Truhlar et al., 2015).

Normothermic CPR can provide ∼30% of pre-arrest cardiac output (CO) (Wik et al., 1996; Sunde et al., 1998; Pytte et al., 2006) but is usually considered discontinued after 20–30 min if the return of spontaneous circulation is not achieved, as these patients show poor clinical outcome (Torke et al., 2015; Perkins et al., 2015a, b). Preclinical studies (Wik et al., 1996; Gervais et al., 1997; Schwarz et al., 2002; Tomte et al., 2010; Welbourn and Efstathiou, 2018) also support this clinical notion, but the maximum duration of CPR in normothermic patients has so far not been determined (Welbourn and Efstathiou, 2018).

It is well documented that cooling with spontaneous circulation slows metabolic rate and, therefore, reduces CO in parallel with reductions in global O_2_ transport (DO_2_) and O_2_ consumption (VO_2_) (Black et al., 1976; Kondratiev et al., 2006; Valkov et al., 2019). In addition, low core temperature *per se* prolongs end-organ survival during hypothermic CPR (Gilbert et al., 2000; Mark et al., 2012; Wanscher et al., 2012). In an intact pig model, we validated these findings and also documented that O_2_ transport (Filseth et al., 2010) and tissue blood flow (Valkov et al., 2019) are maintained during 1–3 h of stable hypothermia (25°–27°C) as well as during rewarming. However, to date, the effects of prolonged (hours) CPR on global DO_2_ and organ blood perfusion have not been assessed in an intact hypothermic animal model. A report from 1986 documented that CPR at 28°C replaced normothermic CO during spontaneous circulation by only 7% after 20 min (Maningas et al., 1986). Subsequent pharmacologic studies on pigs (Krismer et al., 2000; Kornberger et al., 2001) reported the maintenance of coronary perfusion pressure at 28°C during short-lasting CPR, but neither of these studied applied the latest standard CPR algorithm (Truhlar et al., 2015).

New devices make it possible to sustain CPR during evacuation and transport for a longer period of time with a limited number of rescuers (Perkins et al., 2015b). UNN (University Hospital of North Norway) is located in a scarcely populated, subarctic catchment area, making flying time for patients with ambulance airplanes typically around 90 min. This makes evacuation and transportation time 3–4 h for accidental hypothermia patients with CA in need of in-hospital rewarming. Under these circumstances, continuous prehospital CPR is often the only treatment option for these patients.

It is well documented that standard CPR can replace CO only to a certain low level. We, here, hypothesize that this level is largely unaffected by core temperature, and thus, cooling with spontaneous circulation will reduce CO to a level, which eventually can be replaced by CPR. Therefore, the aim of this study was to evaluate the value of CPR as part of prehospital interventions for hypothermic patients with CA in more detail using our intact porcine model of CPR instrumented to determine hemodynamics, global DO_2_, VO_2_, and organ blood flow during continuous CPR for 3 h at 27°C core temperature. To study the effects of low temperature only, ischemic episodes were avoided before CPR, and 27°C corresponds to the core temperature of surviving patients after hypothermic CA. To more closely mimic a clinical scenario, the porcine model was equipped with a commercially available automated chest compression device.

## Materials and Methods

### Ethical Approval

The Norwegian Food Safety Authority approved the study (ref. number: 14/56323). Eleven castrated male pigs (wt. 20–29 kg, age 3 months) from NOROC stock were used. On arrival, the animals were acclimated for 2–5 days before the terminal experiment. Animals were fed twice daily, had free access to water at all times, and received humane care in accordance to the Norwegian Animal Welfare Act.

### Anesthesia and Instrumentation

We previously reported the detailed methods for hemodynamic monitoring, immersion cooling, and blood flow measurements using the porcine animal model (Valkov et al., 2019). Briefly, after fasting the animals overnight, premedication was induced by an intramuscular bolus of ketamine hydrochloride (20 mg kg^–1^), midazolam (30 mg), and atropine (1 mg), and anesthesia was induced by a bolus infusion of fentanyl (10 μg kg^–1^) and pentobarbital-sodium (10 mg kg^–1^) in an ear vein. After tracheotomy and intubation, the animals were connected to a respirator (Siemens Servo 900D, Solna, Sweden), adjusted to maintain PaO_2_ > 10 kPa and PaCO_2_ at 4.5–6.0 kPa uncorrected for temperature (α-stat management). After instrumentation, animals were randomly assigned to two groups [hypothermic CA and CPR for 3 h (*n* = 7), or normothermic CA and CPR for 45 min (*n* = 4)]. During ventricular fibrillation (VF) and CPR, FiO_2_ was set to 1.0. Infusion of fentanyl (20 μg kg^–1^h^–1^), midazolam (0.3 μg kg^–1^h^–1^), and pentobarbital-sodium (4 mg kg^–1^h^–1^) was continued via a femoral vein catheter. In the hypothermia group, anesthesia was discontinued at 27°C. Microspheres were injected to the left ventricle through a 6F fluid filled pigtail catheter (Cordis Corporation, Miami, FL, United States). Core temperature, CO, and venous and mixed venous blood gases were measured via a 7F Swan–Ganz thermodilution catheter (Edwards Lifesciences LLC, Irvine, CA, United States) positioned in the pulmonary trunk. Thermodilution is described as the gold standard for measuring CO during CPR (Carretero et al., 2010). The tip of another 7F Swan–Ganz thermodilution catheter was positioned in the aortic arch via the left femoral artery to monitor arterial blood gas and mean arterial pressure (MAP) and to collect reference blood samples for the microsphere technique. Urinary output was followed from a 14F urinary bladder catheter introduced via a lower abdominal incision. The animals were given 5,000 IE Heparin and allowed to stabilize for 45 min before the start of the experimental protocol.

### Regional Blood Flow Measurements

To determine organ blood flow, stable isotope-labeled microspheres (BioPAL Inc., Worcester, MA, United States) were injected into the left ventricle (Reinhardt et al., 2001). Simultaneously, a reference blood sample was drawn from the tip of a catheter inserted in the left femoral artery and advanced to the aortic arch to calculate regional blood flow. Blood flow was determined in tissue samples from the brain (temporal lobes), kidneys, liver, heart, small intestine, and stomach based on a technique of neutron activation to analyze microsphere content as already described in detail (Valkov et al., 2019).

### Experimental Protocol

**FIGURE 1:**
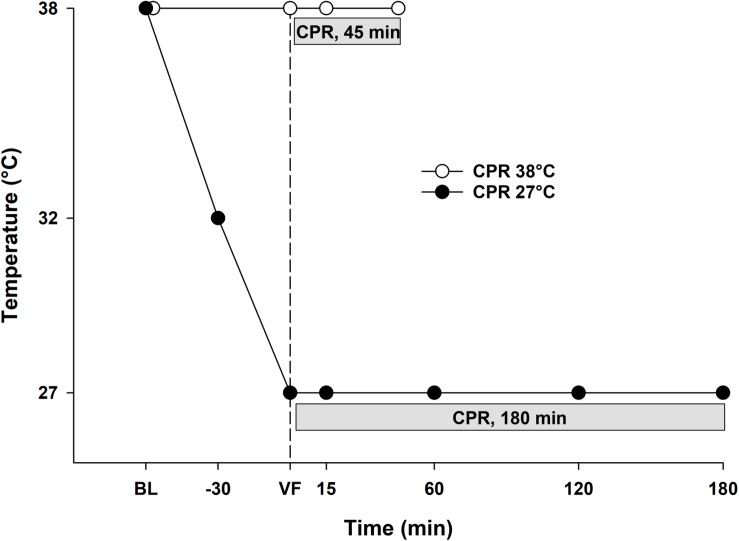
Experimental protocol. 38°C Baseline (BL); Cardiopulmonary resuscitation (CPR); Ventricular fibrillation (VF).

Animals in the hypothermia group were immersion cooled in ice water to a blood temperature of 27°C, and VF was induced by stimulating the epicardial surface using an alternating current (5–20 mA, 6 Hz, and 30 V) conducted via a 15 cm-long needle electrode (Figure 1). The needle was inserted in the epigastric area and pointed toward the heart apex. Correct needle placement was confirmed when aspirating arterial blood from the left ventricle. CA was defined as asystoly or VF on ECG associated with the absence of fluctuation in arterial pressure. After 90 s of CA, an automated chest compression device (LUCAS chest compression system, Physio-Control Inc., Lund, Sweden) was started. The piston on this compression device was equipped with a suction cup to ensure active decompression with a continuous mode compression/decompression duty cycle of 50 ± 5% at a rate of 100 ± 5 compressions/min, and compression depth was 4–5 cm. In the normothermia group, CPR was continued for 45 min, whereas in the hypothermia group, CPR was continued for 3 h.

### Frequency of Measurements

In the normothermia group: during spontaneous circulation at 38°C before VF, and at 15 and 45 min during CPR. In the hypothermia group: during spontaneous circulation at 38°C, during cooling at 32° and 27°C before VF, and during CPR at 15 min and hourly thereafter. Simultaneously, we recorded electrocardiogram (standard leads), heart rate (HR), MAP, central venous pressure (CVP), and urine output, and microspheres were injected. Blood gases were analyzed by simultaneous sampling from arterial, central venous, and mixed venous blood.

### Statistics

Statistical analyses were performed using SigmaPlot statistical software version 14 (Systat Software Inc., Richmond, CA, United States). Normal distribution was checked using the Shapiro–Wilk test. Comparisons between normothermia and hypothermia groups as well as intragroup comparisons at 38°C baseline, 15 min, and 45/60 min of CPR were performed by two-way repeated measures ANOVA. Where significant differences were found, all pairwise multiple comparison procedures were done using the Holm–Sidak test. To compare values within the hypothermia group, we used one-way repeated measures ANOVA for normal distributed variables, and Friedman repeated measures ANOVA on ranks for non-normal distributed variables. Where significant differences were found, Dunnett’s test was used to compare all values within the hypothermia group vs. 38°C baseline, as well as values obtained during CPR at 60, 120, and 180 min vs. CPR at 15 min. The level of significance was set at *p* < 0.05. Data are presented as means and SD.

## Results

No statistically significant differences were found between groups in any of the variables recorded at the start of the experiments. Animals in both groups had spontaneous circulation before induction of VF. Stability of the actual pig model related to hemodynamic function and organ blood flow at normothermia has previously been documented in experiments lasting up to 7 h (Filseth et al., 2010; Valkov et al., 2019). Multiple costal fractures occurred in all animals after CPR, and after 3 h of CPR also sternal fractures were observed in six out of seven animals.

### Comparing Effects of CPR for VF After 15 and 45/60 min, at the Two Different Core Temperatures, 38° and 27°C

#### Hemodynamic Variables

##### 38°C.

**FIGURE 2:**
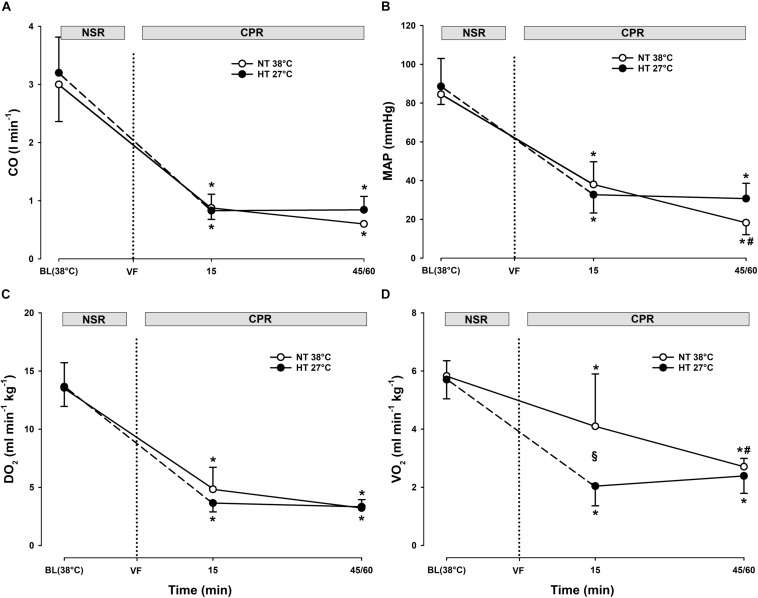
Hemodynamic variables, and global O_2_ transport and O_2_ uptake during CPR at normothermia and hypothermia. **(A)** Cardiac output (CO), **(B)** mean arterial pressure (MAP), **(C)** global oxygen delivery (DO_2_), and **(D)** global oxygen consumption (VO_2_). CPR at normothermia (NT 38°C, *n* = 4), CPR at hypothermia (HT 27°C, *n* = 7). 38°C baseline (BL) (38°C); normal sinus rhythm (NSR); cardiopulmonary resuscitation (CPR); ventricular fibrillation (VF). Comparisons between groups and intragroup comparisons at 38°C baseline, 15 and 45/60 min CPR were done using two-way repeated measures ANOVA and pairwise multiple comparison using Holm–Sidak test. Values are mean ± SD. *p < 0.05 vs. intragroup 38°C baseline; ^#^*p* < 0.05 vs. intragroup 15 min of CPR; ^§^ *p* < 0.05 vs. corresponding value between groups.

Compared to spontaneous circulation at 38°C, after CPR for 15 min at this temperature, CO was significantly reduced from 3.0 ± 0.6 to 0.9 ± 0.2 L min^–1^ (-71%) and MAP was significantly reduced from 85 ± 5 to 38 ± 12 mmHg (-55%) (Figures 2A,B). After 45 min of CPR at 38°C, CO and MAP ended up being reduced by 80 and 78% (0.6 ± 0 L min^–1^ and 18 ± 6 mmHg), respectively, but the reduction in MAP was statistically significant.

##### 27°C.

Compared to spontaneous circulation at 38°C, after CPR for 15 min at 27°C, CO was significantly reduced from 3.2 ± 0.6 to 0.8 ± 0.1 L min^–1^ (-74%) and MAP from 89 ± 14 to 33 ± 9 mmHg (-63%). After 60 min of CPR at 27°C, CO and MAP were reduced by 74 and 65% (0.8 ± 0.2 L min^–1^ and 31 ± 8 mmHg), respectively.

##### Comparisons.

No significant differences between groups in CO or MAP were found after 15 min of CPR. In the 38°C group, MAP after 45 min CPR was significantly lower than MAP after 60 min of CPR in the 27°C group. This is in essential contrast to the 27°C group where both MAP and CO remained unchanged during 60 min of CPR.

#### Oxygen Transport and Extraction

##### 38°C.

**FIGURE 3:**
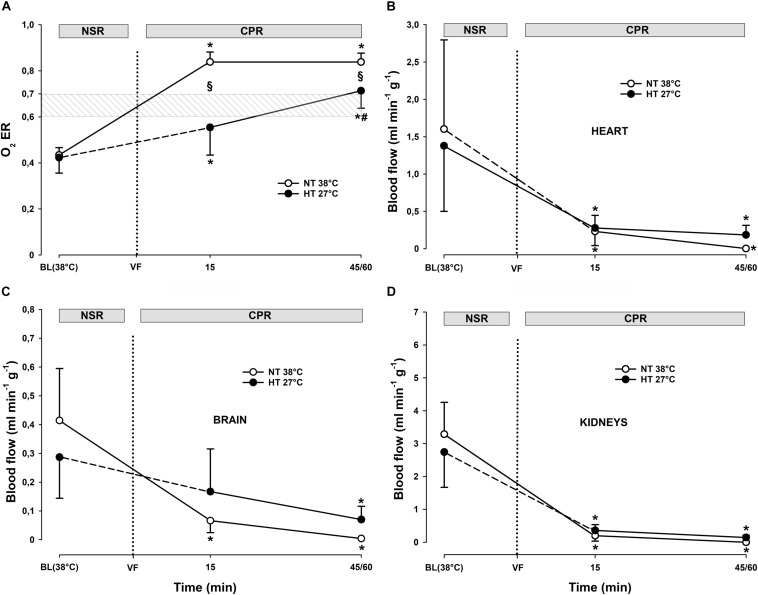
Oxygen extraction ratio, and regional blood flow during CPR at normothermia and hypothermia. **(A)** Oxygen extraction ratio (O_2_ ER), **(B–D)** regional blood flow in the heart, brain, and kidneys. CPR at normothermia (NT 38°C, *n* = 4), CPR at hypothermia (HT 27°C, *n* = 7). 38°C baseline (BL) (38°C); normal sinus rhythm (NSR); cardiopulmonary resuscitation (CPR); ventricular fibrillation (VF). Comparisons between groups and intragroup comparisons at 38°C baseline, 15 and 45/60 min of CPR were done using two-way repeated measures ANOVA and pairwise multiple comparison using the Holm–Sidak test. Values are mean ± SD. *p < 0.05 vs. intragroup 38°C baseline; ^#^*p* < 0.05 vs. intragroup 15 min of CPR; ^§^*p* < 0.05 vs. corresponding value between groups.

**FIGURE 4:**
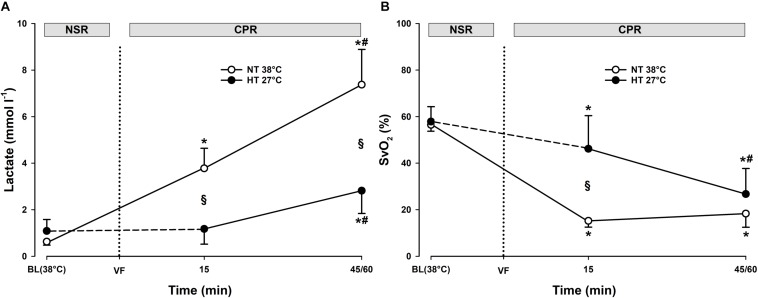
Lactate and mixed venous oxygen saturation during CPR at normothermia and hypothermia. **(A)** Lactate and **(B)** mixed venous oxygen saturation (SvO_2_). CPR at normothermia (NT 38°C, *n* = 4), CPR at hypothermia (HT 27°C, *n* = 7). 38°C baseline (BL) (38°C); normal sinus rhythm (NSR); cardiopulmonary resuscitation (CPR); ventricular fibrillation (VF). Comparisons between groups and intragroup comparisons at 38°C baseline, 15 and 45/60 min CPR were done using two way repeated measures ANOVA and pairwise multiple comparison using the Holm–Sidak test. Values are mean ± SD. *p < 0.05 vs. intragroup 38°C baseline; ^#^*p* < 0.05 vs. intragroup 15 min of CPR; ^§^*p* < 0.05 vs. corresponding value between groups.

Compared to during spontaneous circulation at 38°C, after 15 min of CPR, DO_2_ decreased significantly from 13.6 ± 2.2 to 4.8 ± 1.9 ml min^–1^ kg^–1^ (-64%), and VO_2_ from 5.8 ± 0.5 to 4.1 ± 1.8 ml min^–1^kg^–1^ (-30%) (Figures 2C,D). Accordingly, the O_2_ extraction ratio (ER; DO_2_/VO_2_) (Figure 3A) was significantly increased after 15 min of CPR (from 0.43 ± 0.03 to 0.84 ± 0.04), exceeding the reported critical extraction ratio (ER_crit_) value (0.6–0.7) to provide aerobic metabolism (Leach and Treacher, 1992). After 45 min, ER remained significantly elevated (0.84 ± 0.04). Simultaneously, a reduction in mixed venous O_2_ saturation (SvO_2_) (Figure 4B) from 56 ± 3% during spontaneous circulation to 15 ± 3 and 18 ± 6% after 15 and 45 min of CPR, respectively, took place in parallel with a significant increase in the serum lactate level (Figure 4A), from 0.63 ± 0.15 during spontaneous circulation to 3.78 ± 0.87 after 15 min of CPR increasing to 7.38 ± 1.51 mmol L^–1^ after 45 min of CPR, underlining the existence of inadequate O_2_ transport throughout the last 30 min of CPR at 38°C.

##### 27°C.

Compared to during spontaneous circulation at 38°C, after 15 min of CPR at 27°C, DO_2_ decreased significantly from 13.7 ± 1.7 to 3.6 ± 0.8 ml min^–1^ kg^–1^ (-73%), and VO_2_ from 5.7 ± 0.7 to 2.0 ± 0.7 ml min^–1^kg^–1^ (-64%) (Figures 2A,B). This caused a significant increase in ER from 0.42 ± 0.07 to 0.55 ± 0.12%, but this value is below ER_crit_ (0.6–0.7) (Figure 3A). Simultaneously, SvO_2_ (Figure 4B) fell from 58 ± 6 at 38°C to 46 ± 14 after 15 min of CPR, and after 60 min of CPR, SvO_2_ was reduced to 27 ± 11%, and lactate (Figure 4A) increased after 15 min and 60 min of CPR from 1.17 ± 0.65 to 2.81 ± 0.98 mmol L^–1^, respectively.

##### Differences

Owing to the low core temperature, VO_2_ in the 27°C group was significantly lower than VO_2_ in the 38°C group, whereas no differences between groups in DO_2_ were found. The reduction in SvO_2_ was significantly lower in the 27°C group than in the 38°C group. Also, the increase in lactate in the 27°C group was significantly lower than in the 38°C. Taken together, the higher the SvO_2_, the modest increase in lactate, and the less increase in ER during 60 min of CPR in the 27°C group indicates a better O_2_ transport than in the 38°C group.

#### Organ Blood Flow

Already after 15 min of CPR in the 38°C group, there was a statistically significant reduction in blood flow to the brain, heart, kidneys, stomach, liver, and small intestine (Figures 3B,D). After 45 min of CPR, practically no organ blood flow was detectable. A similar reduction in organ blood flow was measured after 15 min of CPR in the 27°C group, but in essential contrast to the 38°C group, organ blood flow was maintained at this reduced level throughout 60 min of CPR.

### Cooling to 27°C With Spontaneous Circulation

#### Hemodynamic Variables

**FIGURE 5:**
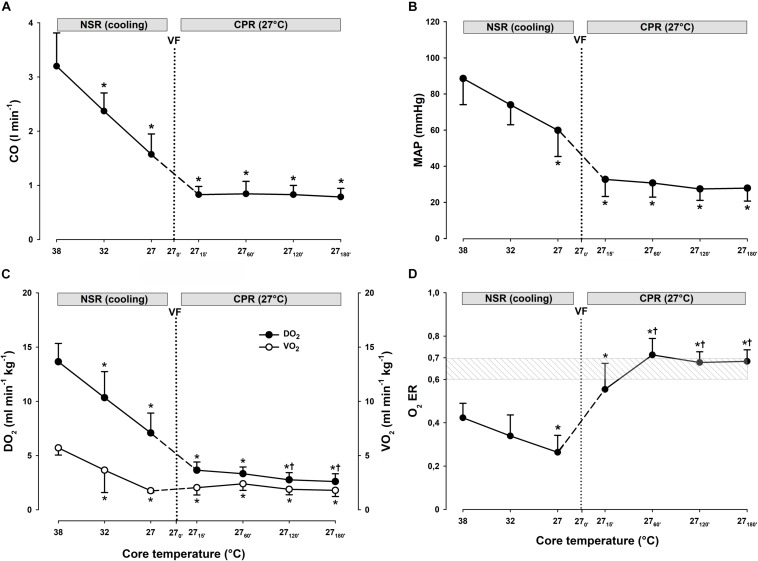
Hemodynamic function, global O_2_ transport, global O_2_ uptake, and O_2_ extraction ratio during cooling and 3 h of CPR at 27°C (*n* = 7). **(A)** Cardiac output (CO), **(B)** mean arterial pressure (MAP), **(C)** global oxygen delivery (DO_2_), and global oxygen consumption (VO_2_), and **(D)** oxygen extraction ratio (O_2_ ER). Normal sinus rhythm (NSR); cardiopulmonary resuscitation (CPR); ventricular fibrillation (VF). To compare values within groups, we used one-way repeated measures ANOVA for normal distributed variables and Friedman repeated measures ANOVA on ranks for non-normal distributed variables. Dunnett’s test was used to compare all values obtained vs. 38°C baseline and also for values obtained during CPR vs. corresponding values after CPR for 15 min. Values are mean ± SD. *p < 0.05 vs. intragroup 38°C baseline; ^†^*p* < 0.05 vs. intragroup 15 min of CPR at 27°C.

Compared to corresponding values at 38°C, cooling with maintained spontaneous circulation gave an almost linear reduction in CO and MAP (Figures 5A,B). At 27°C, CO was significantly reduced from 3.2 ± 0.6 to 1.6 ± 0.4 L min^–1^ (-50%), and MAP significantly reduced from 89 ± 14 to 60 ± 14 mmHg (-33%).

#### Oxygen Transport and Extraction

Global DO_2_ and VO_2_ (Figure 5C) were significantly reduced from 13.7 ± 1.7 to 7.1 ± 1.8 ml min^–1^ kg^–1^ (-48%), and from 5.7 ± 0.7 to 1.8 ± 0.2 ml min^–1^ kg^–1^ (-68%), respectively, giving an ER (Figure 5D) of 0.26, which is far below the ER_crit_ of 0.6–0.7 (Leach and Treacher, 1992).

#### Organ Blood Flow

**FIGURE 6:**
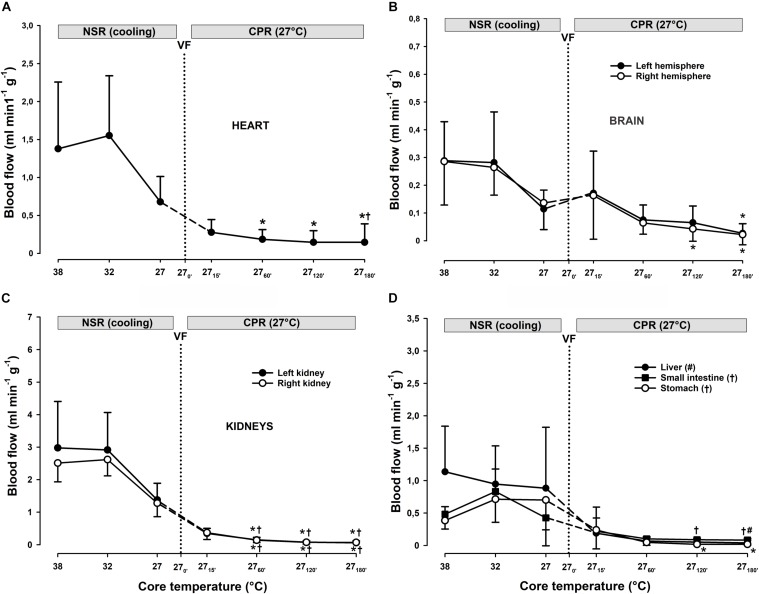
Regional blood flow during cooling and 3 h CPR at 27°C (*n* = 7). **(A–D)** Regional blood flow. Normal sinus rhythm (NSR); cardiopulmonary resuscitation (CPR); ventricular fibrillation (VF). To compare values within groups, we used one-way repeated measures ANOVA for normal distributed variables, and Friedman repeated measures ANOVA on ranks for non-normal distributed variables. Dunnett’s test was used to compare all values obtained vs. 38°C baseline and also for values obtained during CPR vs. corresponding values after CPR for 15 min. Values are mean ± SD. *p < 0.05 vs. intragroup 38°C baseline; ^#^, ^†^*p* < 0.05 vs. intragroup 15 min of CPR at 27°C.

During cooling to 27°C, an apparent reduction in blood flow to all organs measured took place, but these changes did not reach statistical significance (Figure 6).

### Effects of Continuous CPR for VF During 3 h at 27°C

#### Hemodynamic Variables

CPR for 15 min at 27°C gave a significant reduction in CO from 3.2 ± 0.6 to 0.8 ± 0.1 L min^–1^ (-74%), and a significant reduction in MAP from 89 ± 14 to 33 ± 9 mmHg (-63%) when compared to corresponding values during spontaneous circulation at 38°C (Figures 5A,B). Both CO and MAP remained unchanged at these reduced levels throughout the 3 h of CPR at 27°C.

#### Oxygen Transport and Extraction

**FIGURE 7:**
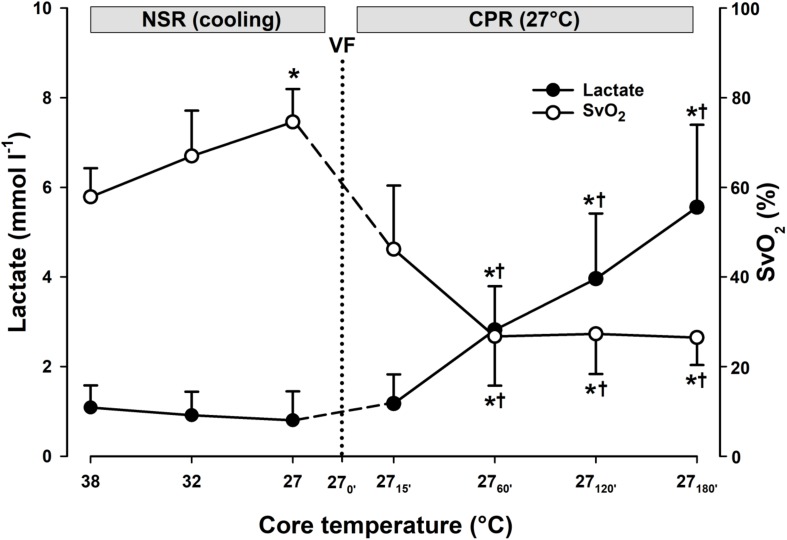
Lactate and mixed venous oxygen saturation during cooling and 3 h of CPR at 27°C (*n* = 7). Mixed venous oxygen saturation (SvO_2_); normal sinus rhythm (NSR); cardiopulmonary resuscitation (CPR); ventricular fibrillation (VF). To compare values within groups, we used one-way repeated measures ANOVA for normal distributed variables and Friedman repeated measures ANOVA on ranks for non-normal distributed variables. Dunnett’s test was used to compare all values obtained vs. 38°C baseline and also for values obtained during CPR vs. corresponding values after CPR for 15 min. Values are mean ± SD. *p < 0.05 vs. intragroup 38°C baseline; ^†^*p* < 0.05 vs. intragroup 15 min of CPR at 27°C.

During 3 h of CPR, the differences between DO_2_ and VO_2_ diminished (Figure 5C), but when compared to their corresponding values after 15 min of CPR at 27°C (7.1 ± 1.8 and 1.8 ± 0.2 ml min^–1^ kg^–1^, respectively), DO_2_ and VO_2_ were further significantly reduced first after 2 h of CPR (2.8 ± 0.7 and 1.9 ± 0.5 ml min^–1^ kg^–1^, respectively). By 1 h of CPR, ER_crit_ (Figure 5D) was approached (0.71 ± 0.08) and remained at or near this critical value over the next 2 h of CPR (0.68 ± 0.05). After 15 min of CPR, SvO_2_ (Figure 7) was reduced from 75 ± 7 to 46 ± 14% followed by a further gradual reduction in SvO_2_ over the next 1 h to 27 ± 11%, but with no change during the next 2 h. After 1 h of CPR at 27°C, a modest but significant elevation of serum lactate from 0.8 ± 0.65 to 2.81 ± 0.98 mmol L^–1^ took place. Serum lactate level increased linearly during the rest of the CPR period reaching 5.56 ± 1.84 mmol L^–1^after 3 h.

#### Organ Blood Flow

Compared to after 15 min of CPR at 27°C, blood flow to the heart remained unaltered during the first 2 h of CPR (Figure 6A). A significant reduction in blood flow to the heart from 0.3 ± 0.2 to 0.1 ± 0.2 ml min^–1^ g^–1^ was found first after 3 h of CPR. Blood flow to both brain hemispheres (Figure 6B) remained unaltered after 1 h of CPR when compared to flow after 15 min of CPR, but flow to both left hemispheres was significantly reduced after 2 h of CPR. First, after 3 h of CPR blood flow to both hemispheres was significantly reduced compared to 15 min of CPR: left hemisphere from 0.16 ± 0.16 to 0.02 ± 0.02 and right hemisphere from 0.17 ± 0.15 to 0.03 ± 0.03, all values in ml min^–1^g^–1^. Blood flow to kidneys (Figure 6C) was significantly reduced already after 1 h of CPR, liver blood flow was significantly reduced after 3 h, whereas flow to the small intestine and stomach was reduced after CPR for 2 and 3 h (Figure 6D).

## Discussion

This experiment, comparing hemodynamic function, O_2_ transport, and organ blood flow during spontaneous circulation at 38°C, and during subsequent CPR for VF at 38°C or 27°C, demonstrated that after 15 min of CPR, these variables were generated at the same reduced levels irrespective of the core temperature. This corresponds with our working hypothesis. Continued CPR for a total of 3 h at 27°C maintained MAP and CO, blood flow to the brain and heart at the same reduced level, and provided adequate DO_2_ to support aerobic metabolism. The results also indicate the presence of a compensatory autoregulation of organ blood flow during hypothermic CPR with critically reduced circulation. By contrast, after 45 min of CPR at 38°C, MAP was significantly reduced, and blood flow to vital organs became undetectable in parallel with derangements in organ metabolism.

### Cardiopulmonary Resuscitation

The European Resuscitation Council guidelines (2015) (Truhlar et al., 2015) recommend that in accidental hypothermia patients with CA, CPR should be started immediately and continued during evacuation and transport. These guidelines also advocate using mechanical chest compression devices in special scenarios, e.g., during rescue of hypothermic patients in a moving ambulance, even though it is still debated if compression devices are superior to manual compression for CA (Rubertsson and Karlsten, 2005; Aufderheide et al., 2011; Putzer et al., 2013).

Several studies using the pig as experimental model have explored the effects of different techniques for CPR on pressure generation and organ blood flow during normothermia, with and without the aid of compression devices (Wik et al., 1996; Sunde et al., 1997, 1998; Langhelle et al., 2002; Schwarz et al., 2002; Pytte et al., 2006; Tomte et al., 2010; Karlis et al., 2014). Alternative techniques most often tested are active compression–decompression CPR (ACD-CPR) with and without use of an impedance threshold valve CPR (ITV-CPR) in comparison to standard CPR (Langhelle et al., 2002; Schwarz et al., 2002). These studies consistently reported better pressure end flow generation during ACD-CPR, attributed the elevated negative intrathoracic pressure during active decompression facilitating better filling of the heart between compressions. Mechanical chest compression devices most often offer ACD-CPR, as used in the present experiment, but guidelines do not consider ACD-CPR superior to good standard CPR (Wenzel et al., 2010).

From clinical practice and preclinical experiments, we know that the effectiveness of normothermic CPR to maintain blood flow rapidly deteriorates. In the present experiment, hemodynamics and organ blood flow were largely maintained during the first 2 h of hypothermic CPR. After that, a modest reduction in these variables was accompanied with a small increase in lactate production, but far from ending in a circulatory collapse as demonstrated in normothermic animals after 45 min of CPR.

### Hemodynamics and Blood Flow

In pig models of normothermic CPR, hemodynamic and organ blood flow data are well documented after 5–10 min of CPR (Sunde et al., 1997, 1998; Langhelle et al., 2002; Steen et al., 2002). The present study evaluated the effects of CPR after 15 min, and the actual hemodynamic results are comparable to those reported by Carretero et al. (2010) after 15 min and by Steen et al. (2002) after only 5 min of CPR. Similar to our results, Gervais et al. (1997) reported almost complete cessation of myocardial and cerebral blood flow after 45 min of normothermic CPR.

### Oxygen Transport

With compromised circulation, VO_2_ will eventually become dependent on the DO_2_. The O_2_ extraction ratio (ER), the ratio between VO_2_ and DO_2_, can be used to demonstrate this physiologic mechanism. With limited O_2_ supply, ER approaches a critical value, ER_crit_, where tissue O_2_ consumption is ruled by O_2_ supply (Schumacker et al., 1987), reported to be 0.6–0.7 (Leach and Treacher, 1992) at normothermia. During hypothermia, the ER_crit_ is unchanged (∼0.65) (Schumacker et al., 1987), even with an increase in vascular resistance, which may override the physiologic local metabolic feedback to arterioles to regulate blood flow (Schumacker et al., 1987). During spontaneous circulation at 27°C, the reduced metabolism caused a significantly reduced VO_2_ in parallel with the reduced DO_2_. Importantly, we found that during the first 2 h of CPR at 27°C, despite the 74% reduction in CO, DO_2_ was maintained at the same low level as during spontaneous circulation. Beyond 2 h of CPR, a significant fall in DO_2_ took place, followed by a moderate, yet significant, increase in lactate production and a fall in SvO_2_. For comparison, also in our previous pig experiment mentioned with 3 h of spontaneous circulation at 27°C, we found an elevation of ER to a level approaching ER_crit_ indicating a marginal O_2_ supply during hypothermia, but this marginal supply was followed by a successful rewarming (Valkov et al., 2019).

Taken together, the actual values for lactate production, SvO_2_, and ER demonstrate that 3 h of CPR at 27°C provides marginal, but sufficient, O_2_ transport for aerobic metabolism in vital organs. Importantly, we also found that VO_2_ was maintained during 3 h of CPR, indicating a use-dependent O_2_ consumption. This is in essential contrast to normothermic CPR where DO_2_ after 15 min was unable to provide aerobic metabolism, reflected by the significant elevation of ER exceeding the ER_crit_ value, in parallel with a substantial elevation in serum lactate and lowering of SvO_2_.

## Limitations

The porcine model was chosen in our experiment because of its previous use in exploring the effects of CPR, especially when using a compression device (Steen et al., 2002; Rubertsson and Karlsten, 2005). However, the incidence of fractures of the sternum and multiple costa after 3 h of CPR in all our animals clearly demonstrates a major limitation when applying equipment designed for humans. A multicenter survey has documented the absence of fatal injuries in patients after conventional CPR using a compression device (Smekal et al., 2014), but little is known about damage to the thoracic skeleton or to the heart of patients after prolonged mechanical chest compressions.

### Potential Translational Value

From a clinical perspective, to date, the only possible way to treat patients with continuous CPR during evacuation and transport in an ambulance car is by using an automated chest compression device. Therefore, to treat accidental hypothermia patients with cardiac arrest in our catchment area, we aim to equip all car ambulances with automated compression devices. Further, the beneficial physiologic effects of prolonged CPR for cardiac arrest at reduced core temperatures, emerges to build a new basis with unknown potential for patient survival after the ensuing in-hospital rewarming. From clinical reports already listed, extracorporal circulation has evolved to become the method of choice for rewarming these patients. Preferentially, extra corporal membrane oxygenation (ECMO) is increasingly being used. By applying ECMO for rewarming, cardio/respiratory support can be continued during an ensuing ICU stay for days, if needed. This is to treat rewarming injury, which often manifests itself as cardiac dysfunction caused by hypothermia/rewarming, in addition to the unknown consequences of prolonged CPR to cause physical damage to the heart, lungs, and the thoracic skeleton. However, although effects of extracorporal rewarming to increase survival seems promising, more experimental and clinical work is needed to customize this method for rewarming patients from accidental hypothermia.

## Conclusion

The results of this study support our working hypothesis and show the favorable effects of CPR when given at 27°C in comparison to normothermic CPR. The beneficial effects of CPR in providing a limited, but adequate, DO_2_ during a 3 h period at 27°C, combined with an apparent patent peripheral circulatory function, make continuous CPR the most valuable prehospital intervention as a bridge for hypothermic patients to receive in-hospital rewarming.

## Data AvailabilIty Statement

The raw data supporting the conclusions of this article will be made available by the authors, without undue reservation, to any qualified researcher.

## Author Contributions

JN, TT, GS, and TK contributed to the conception and design. JN, SV, RM, TS, TN, and TK contributed to the completion of experiments and collection of data. TT, GS, TK, JN, SV, RM, and TS contributed to the data analysis and interpretation. JN, TT, and GS contributed to the drafting the manuscript for intellectual content. JN, SV, RM, TS, TK, TN, GS, and TT contributed to the revision of the manuscript.

## Conflict of Interest

The authors declare that the research was conducted in the absence of any commercial or financial relationships that could be construed as a potential conflict of interest.

## Abbreviations

ACD-CPR: active compression–decompression
CA: cardiac arrest
CPR
ITV-CPR: impedance threshold valve CPR
CPR: cardiopulmonary resuscitation
CVP: central venous pressure
DO_2_: global oxygen transport
ER O_2_: extraction ratio
HR: heart rate
MAP: mean arterial pressure
VO_2_: oxygen uptake
CO: cardiac output
VF: ventricular fibrillation.
